# Elimination of Drifts in Long-Duration Monitoring for Apnea-Hypopnea of Human Respiration

**DOI:** 10.3390/s16111779

**Published:** 2016-10-25

**Authors:** Peng Jiang, Rong Zhu

**Affiliations:** State Key Laboratory of Precision Measurement Technology and Instrument, Department of Precision Instrument, Tsinghua University, Beijing 100084, China; jiang-p13@mails.tsinghua.edu.cn

**Keywords:** humidity drift, wavelet analysis, sleep apnea, hot-film airflow sensor

## Abstract

This paper reports a methodology to eliminate an uncertain baseline drift in respiratory monitoring using a thermal airflow sensor exposed in a high humidity environment. Human respiratory airflow usually contains a large amount of moisture (relative humidity, RH > 85%). Water vapors in breathing air condense gradually on the surface of the sensor so as to form a thin water film that leads to a significant sensor drift in long-duration respiratory monitoring. The water film is formed by a combination of condensation and evaporation, and therefore the behavior of the humidity drift is complicated. Fortunately, the exhale and inhale responses of the sensor exhibit distinguishing features that are different from the humidity drift. Using a wavelet analysis method, we removed the baseline drift of the sensor and successfully recovered the respiratory waveform. Finally, we extracted apnea-hypopnea events from the respiratory signals monitored in whole-night sleeps of patients and compared them with golden standard polysomnography (PSG) results.

## 1. Introduction

Sensor drift is a common phenomenon that exists in biomedical electric recordings [1]. A gradual change in any quantitative characteristics induced by environment variation, such as temperature and humidity, is called drift. Drifts usually bring about a lot of problems. For example, electrocardiographs (ECG), electroencephalographs (EEG), and electromyograms (EMG) are sometimes affected by perspiration [2]; a photoplethysmogram (PPG) also suffers with a baseline drift due to activity or respiration [3]. Temperature and humidity may arouse chemical gas sensor drift [4]. To overcome sensor drifts, a commonly used method is done through complicated calibration measurements and conducting real-time compensation with the aid of temperature or humidity sensors [5]. However, the calibration method is not valid in some cases when the environment variation cannot be detected accurately. It is necessary to find out alternative effective methods to eliminate drifts, simultaneously retain desired characteristic signals, and ultimately execute feature extraction. 

Physiological signals are typically non-stationary and complex. Interference sources are always multifold and unstable as well. The human respiratory signal is a vital physiological signal. Sleep apnea is an involuntary cessation of breathing that occurs while the patient is asleep [6]. Obstructive sleep apnea-hypopnea syndrome (OSAHS) directly relates to human respiration, which is suffered by 20%–40% of elderly people, and more than half of patients remain undiagnosed [7]. As a matter of fact, sleep apnea may strike anyone at any age, even children [8]. In order to diagnose OSAHS, a system called polysomnography (PSG) [9] is commonly used in professional sleep labs and operated by physicians, which is relatively inconvenient and expensive. Consequently, a portable monitor (PM) has been proposed as a substitute for the PSG in pre-diagnostic assessment on patients with suspected OSAHS by using micro airflow sensors [10,11,12,13]. Respiratory airflow is an important parameter for diagnosing OSAHS. However, human exhaled gas contains many water vapors (RH > 85%) [14]. The water vapors gradually condense on the surface of the respiratory sensors to form a thin water film and thus lead to a significant sensor drift in long-duration respiratory monitoring. To eliminate respiration sensor drifts, effective methods based on features of physiological signals are necessary.

As mentioned above, an uncertain drift generally exists in respiratory sensor outputs due to variations of airflow temperature and humidity. An effective method to eliminate baseline drifts is necessary. To realize temperature compensation, we have proposed to use a constant-temperature difference (CTD) mode to operate the hot-film sensor [15,16]. To eliminate humidity-induced baseline drift in the respiratory signals, we propose to use the discrete wavelet transform (DWT) approach in this paper. Wavelet transform allows not only frequency but also time localization, and enables us to analyze nonlinear and nonstationary signals [17]. Wavelet signal processing can represent signals sparsely, captures the transient features of signals, and enables signal analysis at multiple resolutions, which has advantage in processing respiration signals.

**Figure 1:**
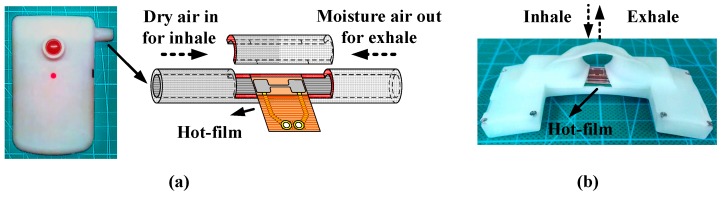
(**a**) Hot-film installation in a tube for respiration monitoring; (**b**) Hot-film installation on surface of sensing strip adhered underneath nostril.

We developed a micro hot-film airflow sensor for monitoring OSAHS [15,16]. The micro-sensor was installed in a pipeline [15] or adhered on a sensing strip underneath a human nostril [16] to detect respiratory airflows. Figure 1 shows two different installation modes for respiration monitoring. Using either of the modes, we observed a significant baseline drift existing in the sensor signals when monitoring respiration over a whole night’s sleep, which greatly damaged apnea-hypopnea extraction. In this paper, we propose an advanced algorithm to eliminate sensor baseline drifts and reconstruct respiratory waveforms. Finally, we used the monitor with the advanced method to extract apnea-hypopnea events from the respiratory signals detected in sleeps of OSAHS patients and compared them with the PSG results to validate the effectiveness.

## 2. Mechanism Analysis for Humidity Drift

### 2.1. Hot-Film Airflow Sensor

**Figure 2:**
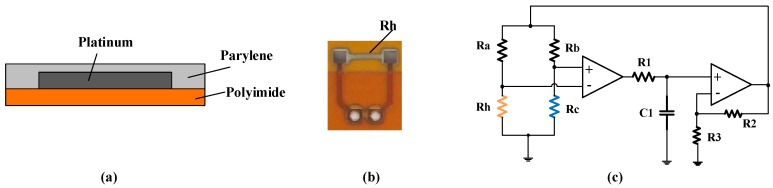
(**a**) Configuration of hot-film sensor; (**b**) Sensor prototype; (**c**) CTD circuit.

The hot-film flow sensor is composed of a platinum thin film fabricated on a flexible polyimide substrate by using micromachining deposition [15,16], shown in Figure 2a,b. A nanoscale thick parylene film was deposited on the sensor surface and served as an encapsulation for waterproof and dustproof characteristics. The measuring principle of the hot-film flow sensor is based on convective heat transfer. The sensor is operated in a CTD mode, which keeps the temperature difference (ΔT) of the hot-film from the ambient temperature constant while changing the heating voltage. The temperature difference ΔT is set as about 10–30 °C for respiration detection. The hot-film flow sensor operated in the CTD mode has a fast dynamic response and high sensitivity due to using feedback to provide the automatic adjustment of electrical compensation [15].

Figure 2c shows a CTD circuit diagram of the sensor. *R_h_* represents a hot-film resistor, *R_c_* is a temperature compensation resistor, *R_a_* and *R_b_* are the balance resistors. Based on the CTD principle and King’s law [18], the output voltage U of the hot-film flow sensor can be formulated by

(1) $$U^{2}=R_{h}{\left(1+\beta \right)}^{2}\cdot \Delta T\cdot \left(A+B\sqrt[{n}]{V}\right)$$

where *V* is the airflow velocity, $\beta =R_{a}/R_{h}$. *A* and *B* are the constant parameters depending on the fluid properties and geometric features of the hot-film, *n* is a geometric factor; $A\propto \lambda \cdot {\left(\frac{C_{p}}{\mu \cdot \lambda }\right)}^{0.2}$ and $B\propto \lambda \cdot \sqrt{\frac{\rho }{\mu }}\cdot {\left(\frac{C_{p}}{\mu \cdot \lambda }\right)}^{0.33}$, where $\rho ,\;\mu ,\;C_{p},\lambda$ are the fluid density, viscosity coefficient, specific heat capacity, and thermal conductivity. It is shown that the baseline output and the scale factor of the airflow sensor are dependent on the fluid properties and the heating source. The fluidic humidity changes the fluid properties and also affects the heat source when the water condenses on the sensor surface, which brings about drifts.

### 2.2. Simulation of Humidity Drift for Hot-Film Flow Sensor

After long exposure duration in a high humidity environment, e.g., human exhaled air, the water vapor gradually condenses on the surface of the hot-film sensor so as to form a thin water film on the sensor surface. The dynamic behavior of the water film forming on the sensor surface is complicated in respiratory monitoring; the vapor condensation makes the water film gradually thicken, but the thermal diffusion of the hot-film sensor and the inhaled dry air make the water film evaporate. After a long respiratory monitoring duration, the formation of the water film may reach equilibrium. This water film formation affects the temperature distribution around the hot-film, and thereby affects the sensor output, exhibiting a drift over time. To demonstrate the humidity drift, we conducted a numerical simulation to establish a relationship between the condensed water film and the temperature of the hot-film electrically heated by a constant power.

**Figure 3:**
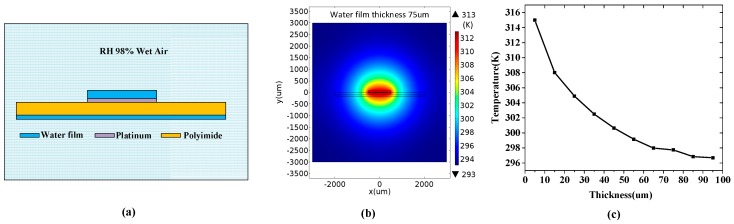
(**a**) Schematic view of hot-film covered by thin water film in 98% RH wet air; (**b**) Simulated temperature distribution around the hot-film; (**c**) Hot-film temperature changing with the thickness of the water film.

Figure 3a shows a schematic view of a hot-film covered with a thin water film in a 98% RH wet air environment. The temperature distribution around the hot-film electrically heated by a constant power (10 mW) was simulated using a non-isothermal flow model in Comsol Multiphysics [19]. In the non-isothermal flow model, there is heat transfer in solids and laminar fluids, and they are coupled with each other. The laminar flow wall was set as a no-slip boundary. The thickness of the water film was set from 5 μm to 100 μm. The ambient temperature was set as 293 K and the ambient moisture air humidity was set to be 98% RH. The simulated temperature distribution is shown in Figure 3b. The extracted hot-film temperature changing with the thickness of the condensed water film is shown in Figure 3c, which demonstrates that the hot-film temperature decreases with the increase of the water film thickness. 

### 2.3. Experiment and Results

Two experiments were conducted to understand the relationship between the humidity and the airflow sensor output. In first experiment, a controllable moist air cabinet was used to establish a humid environment to mimic human respiratory airflow. An ultrasonic humidifier (LifePlus^®^) was used to generate an air humidity that was measured by a hygrometer SHT15 (Sensirion^®^) with a ±2% accuracy in 10%–90% RH and ±3% in 90%–95% RH. A pulse-width-modulation proportional-integral-derivative(PWM-PID) controller was used to keep the environment humidity at a certain constant value. As mentioned before, the water vapors would evaporate simultaneously with the condensation over the sensor surface. The evaporation rate is dependent on the heating temperature of the hot-film and the airflow rate. The condensation rate is dependent on the humidity. When the humidity is lower than a certain value (called the threshold), the evaporation rate is larger than the condensation rate, which means the humidity-induced water film cannot be formed on the sensor surface. When the humidity is higher than the threshold, the condensation rate is larger than the evaporation rate, and the water film accumulates. It means the thickness of the water film condensed on the sensor surface will increase over time. Consequently, sensor drift occurs. 

**Figure 4:**
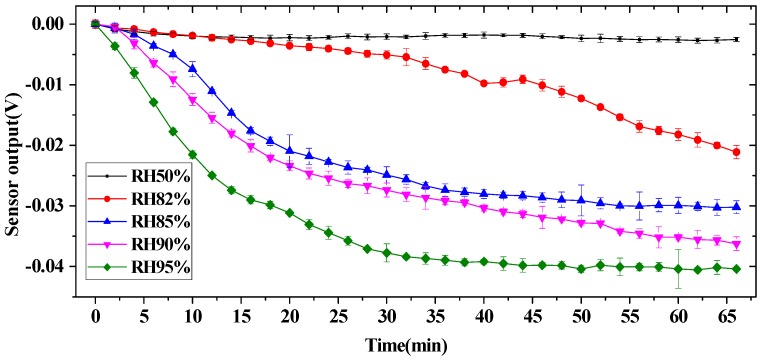
Sensor drift under different humidity environments.

Figure 4 shows the experiment results under different humidity conditions in still air, where error bars representing the standard deviation of uncertainty are provided. The sampling frequency of the respiratory sensor was set as 100 Hz. It was observed that the sensor output decreased over time under a high humidity environment (RH > 82%), whereas the sensor output was comparatively stable under low humidity 50% RH, which indicated that the water film was not formed on the sensor surface due to the evaporation rate being larger than the condensation rate. When the relative humidity was larger than 85%, the sensor output decreased dramatically over time at first and then approached equilibrium, which indicated the water film was gradually thickening and finally approached saturation. The results also show that higher air humidity corresponds to a faster response of the sensor output. Specifically, the response time of the sensor to a higher humidity of 95% RH was about 30 min and over 50 min for 90% RH and 85% RH. 

**Figure 5:**
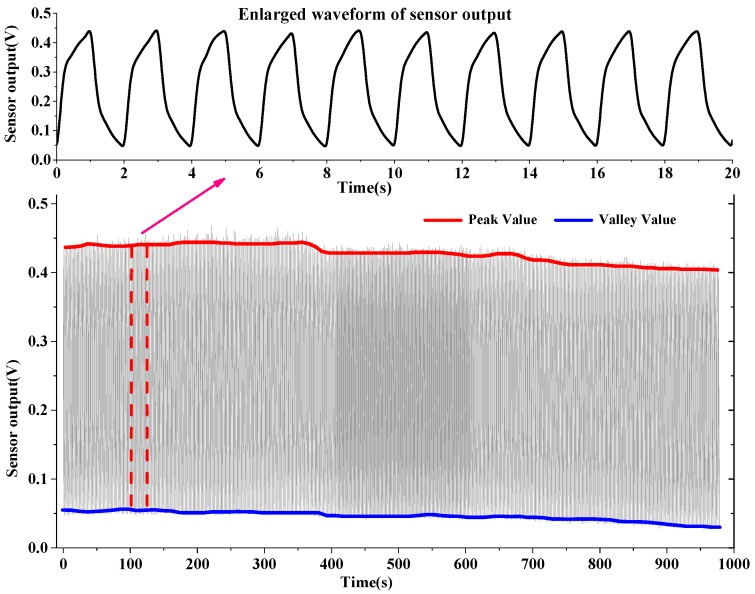
Measurements on alternating airflow mimicking respiratory fluctuation in moist air.

Another measurement on alternating airflow mimicking exhaled and inhaled airflow of human respiration under humidity 98% RH was further conducted by using air pumps to blow the moist airflow into and out of a tube where the hot-film flow sensor was installed. The experiment results are shown in Figure 5, where the peak and valley of the sensor output obviously drift. The valley represents the baseline of the sensor output. The baseline drift deflected the respiratory signals, and brought about a significant error in apnea and hypopnea. Comparatively, the peak-to-peak values of the sensor outputs were less influenced by the humidity. 

In practical application, e.g., in clinical testing, the circumstance is more complicated than that of the above simulation test. The airflow humidity is unsteady and the exhaled breathing gas contains complex compounds which also arouse uncertain baseline drifts in the respiratory sensor outputs.

## 3. Remove Drifts for Respiratory Sensors

### 3.1. Wavelet Signal Processing

**Figure 6:**
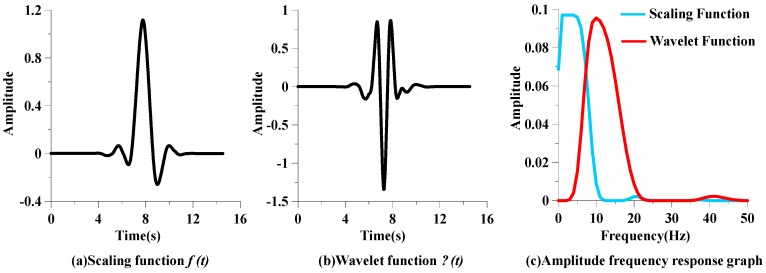
Sym8 scaling function and wavelet function with their amplitude frequency response.

A wavelet is a wave-like oscillation with an amplitude that begins at zero, increases, and then decreases back to zero [20]. Compared with other wavelet effects, the orthogonal Symlet8 (*sym*8) wavelet was selected to remove the baseline drift of the respiration signal and retain the apnea-hypopnea features. The Sym8 wavelet [21] has a scaling function, shown in Figure 6a, and a wavelet function, shown in Figure 6b. The amplitude frequency responses of the scaling and wavelet functions of *sym* are shown in Figure 6c, which reveals the scaling function φ(*t*) can be regarded as a low-pass filter and the wavelet function ψ(*t*) is as a band-pass filter. 

**Figure 7:**
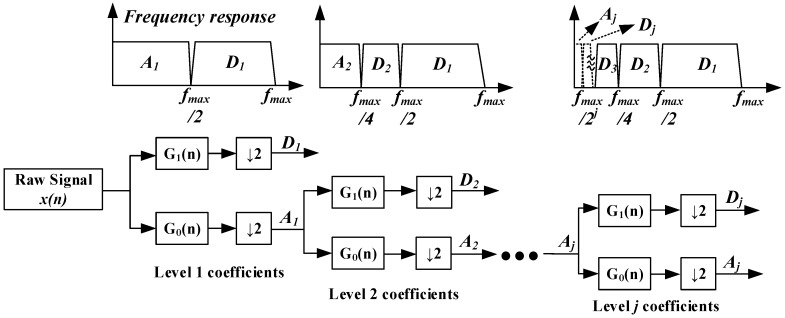
Discrete wavelet transform decomposition processing diagram.

**Figure 8:**
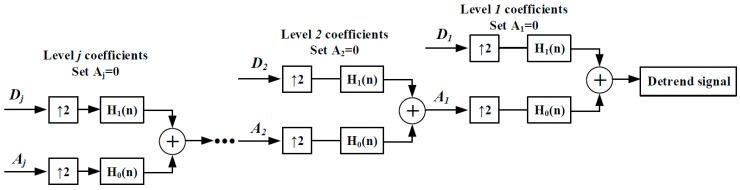
DWT-based reconstructed signal for drift elimination.

In discrete wavelet processing, the raw signal *x*(*n*) goes through the filter banks *G*_1_(*n*) and *G*_0_(*n*). *G*_1_(*n*) and *G*_0_(*n*) are associated with a half-band low-pass filter φ(t) and a half-band high-pass filter ψ(t), as shown in Figure 7. After being filtered by *G*_0_(*n*) and *G*_1_(*n*), half of the samples can be eliminated, which can be seen as the symbol ↓2 in Figure 7. After a cascade decomposition, the raw signal can be calculated to be a series of low-frequency trend signals $A_{j}$ and a series of high-frequency detail signals $D_{j}$. It is also called multi-resolution analysis [22]. The trend signal $A_{j}$ contains all of the sensor drifts. The signal $D_{j}$ contains apnea-hypopnea features. To eliminate low-frequency baseline drift and simultaneously retain apnea-hypopnea features, we set each level $A_{j}$ to zero and then reconstruct each level signal; this process is also called the detrend process. The reconstruction process is just a reverse cascade calculation, as shown in Figure 8. *H*_0_(*n*) and *H*_1_(*n*) are inverse sequences of *G*_0_(*n*) and *G*_1_(*n*), which contain the characteristics of low-pass filter *G*_0_(*n*) and high-pass filter *G*_1_(*n*). After a cascade reconstruction, each level of $A_{j}$ is set to zero and up-sampled by two; each $D_{j}\left(t\right)$ is also up-sampled by 2, as shown in Figure 8. 

Using the discrete wavelet detrend processing algorithm, the baseline drift can be well eliminated from the raw respiratory signals. The key parameters of respiration should be set correctly. Normal respiratory frequency is about 8–24 bpm, i.e., about 0.13–0.4 Hz. According to the fact that a cycle of breath includes one inhale and one exhale process, and the hot-film sensor was not capable of identifying the direction of airflow, and thus the frequency of the detected respiratory signal is the double of the real respiratory frequency, i.e., 0.26–0.8 Hz. Therefore, we set 0.2 Hz as the wavelet cut-off frequency of a high-pass filter. All above processings are operated in LabVIEW software. Compared with the conventional band-pass filter, the above wavelet approach has advantages in signal analysis and feature extraction for complex and unsteady human respiratory signals. The wavelet approach can help us to make a multi-resolution analysis of the signal, and thus we can comprehensively understand the signal features. Furthermore, the wavelet approach can give us a means to reconstruct the signals with specific frequency bands, which can be recognized by the wavelet analysis. For example, it is seen that the detected respiratory signal has a frequency of 0.26–0.8 Hz, and an apnea-hypopnea event contains transient signals in 3–8 Hz. To pick up these features, two frequency bands can be used to reconstruct the respiration in the detrend process, where the low-frequency drift and other disturbances (e.g., body movement) can be eliminated.

**Figure 9:**
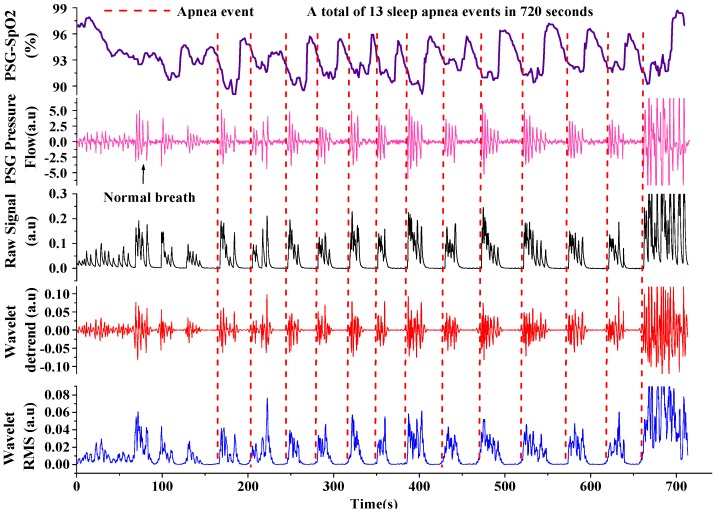
Identification of apnea-hypopnea events from respiratory signals by the wavelet algorithm.

To validate the processing algorithm, we conducted several sleep respiratory monitorings for OSAHS patients using the hot-film respiratory sensor [15] and a PSG (ALICE 5, PHILIPS RESPIRONICS) simultaneously. The experiments on human subjects were approved by the Ethical Review Board of Tsinghua University (No. 20140084). We chose one patient (60 years old, 175 cm height, weight of 84 kg) as an example. The raw respiration signal detected by the hot-film sensor, the oxygen saturation and nasal pressure detected by a PSG, and the processed signals are demonstrated in Figure 9. Normal breath and typical apnea events are marked to show the apnea feature, where the apnea was identified by a respiratory flow drop of 90% for more than 10 s incorporated with 3% oxygen desaturation [23,24].

### 3.2. Extracting Apnea-Hypopnea Events and Results

**Figure 10:**
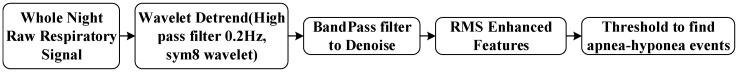
Feature extraction algorithm for apnea and hypopnea events.

Figure 10 shows the signal processing algorithm to extract apnea-hypopnea events from the respiratory signals. Firstly, the raw respiratory signals were processed by using the wavelet detrend processing introduced in Section 3.1 to remove the baseline drift. 

The modified signals were further processed by a band-pass filter to denoise them. Then the root-mean-square (RMS) of the processed signals was calculated by setting a time window with a width of 1 s and used to identify the apnea-hypopnea events according to specific threshold values, such as 50% of the average peak value of pre-event respiration for hypopnea and 10% for apnea [23]. The index of the gold standard PSG served as an indicator to evaluate our device and algorithm. Table 1 lists the apnea-hypopnea index (AHI) figured out from the whole night’s sleep monitoring data of seven OSAHS patients by using our device and PSG at the same time. AHI is an index used to indicate the severity of sleep apnea, which is represented by the number of apnea and hypopnea events occurring per hour during sleep (normal 0–5, mild 5–15, moderate 15–30, severe >30). The comparison results indicate that the effective ratio of the proposed device and algorithm for extracting the apnea-hypopnea events is more than 95%. The current limitation of our study is the insufficience of clinical test for a larger sample of patients, which will be done in our future work. In addition, the methodology will be further optimized in self-adaptability and on-line operations.

## 4. Conclusions

In this paper, we theoretically analyzed and eliminated the baseline drift of the respiration sensor induced by moist respiratory airflow. It has been shown that human exhaled air contains a lot of water vapor, a long exposure duration to which makes leads to respiratory sensor drift. To overcome the humidity-induced baseline drift and extract apnea-hypopnea events from the monitored respiratory signals, a wavelet-based processing algorithm was proposed. The proposed signal processing was used to automatically extract the apnea-hypopnea events from the monitored respiratory signals of OSAHS patients and compared with PSG results. The effective ratio of AHI extraction is more than 95%, which validates the effectiveness of the drift elimination and feature extraction methodology. The proposed wavelet detrend method can be used for eliminating not only humidity drifts but also other drifts induced by elements such as temperature variation, tilt variation of the monitor, etc.

## Figures and Tables

**Table 1:** Apnea-hypopnea events extracted by PSG and the proposed respirometry.

Patients	Infomation		PSG Total Times	Our Device Total Times	AHI (times/h)		Effective Ratio
	Age	Sex			PSG	Device	
Patient 1	31	Female	259	266	37	38	97.3% Good
Patient 2	34	Male	517	507	70	68	98.1% Good
Patient 3	70	Male	115	117	27	27	98.3% Good
Patient 4	55	Female	327	336	54	55	97.2% Good
Patient 5	45	Male	125	129	20	21	96.8% Good
Patient 6	73	Male	356	345	56	54	96.9 % Good
Patient 7	60	Male	393	410	59	61	95.7% Good
